# Reviewer acknowledgement 2014

**DOI:** 10.1186/1546-0096-13-3

**Published:** 2015-02-16

**Authors:** Alberto Martini, Charles Spencer

**Affiliations:** University of Genoa, Genoa, Italy; Ohio State University, Columbus, USA

## Abstract

The Editors of *Pediatric Rheumatology* would like to thank all our reviewers who have contributed to the journal in Volume 12 (2014).

**Brent Adler**

USA

**Amita Aggarwal**

India

**Rachel Annunziato**

USA

**Jordi Anton**

Spain

**Tadashi Ariga**

Japan

**Tadej Avcin**

Slovenia

**Rose Ayoob**

USA

**Judith Barash**

USA

**Alexandre Belot**

France

**Timothy Beukelman**

USA

**John Bohnsack**

USA

**Sharon Bout-Tabaku**

USA

**Paul Brogan**

United Kingdom

**Juergen Brunner**

Austria

**Ruben Burgos-Vargas**

Mexico

**Jon Burnham**

USA

**David Cabral**

Canada

**Scott Canna**

USA

**Joseph Carcillo**

USA

**Jeffrey Chaitow**

Australia

**Gaetano Chirico**

Italy

**Rolando Cimaz**

Italy

**Jacqui Clinch**

United Kingdom

**Laura Coates**

United Kingdom

**Gail Cohen**

USA

**Randy Cron**

USA

**Ruben Cuttica**

Spain

**Rubén José Cuttica**

Argentina

**Michael Davey**

USA

**Marietta de Guzman**

USA

**Fatma Dedeoglu**

USA

**Christopher Denton**

United Kingdom

**Pavla Dolezalova**

Czech Republic

**Fernanda Falcini**

Italy

**Anders Fasth**

Sweden

**Francis Fatoye**

United Kingdom

**Brian Feldman**

Canada

**Polly Ferguson**

USA

**Jill Ferrari**

United Kingdom

**James Galloway**

United Kingdom

**Kathleen Haines**

USA

**Najia Hajjaj-Hassouni**

Morocco

**Liora Harel**

Israel

**Paul Harmatz**

USA

**Philip Hashkes**

Israel

**Christian Hendriksz**

United Kingdom

**Troels Herlin**

Denmark

**Christinae Hermann**

Germany

**José Hernández-Rodríguez**

Spain

**Aimee Hersh**

USA

**Anne Hinks**

United Kingdom

**Mark Hoeltzel**

USA

**Mark Hogan**

USA

**Roger Hollister**

USA

**Annacarin Horne**

Sweden

**Gerd Horneff**

Germany

**Zeng Huasong**

China

**Adam Huber**

Canada

**Birgit Juul-Kristensen**

Denmark

**Shoji Kagami**

Japan

**Sylvia Kamphuis**

The Netherlands

**Kazunari Kaneko**

Japan

**Alfred Kim**

USA

**Susan Kim**

USA

**Marisa Klein-Gitelman**

USA

**Suleyman Koca**

Turkey

**Isabelle Kone-Paut**

France

**Agnieszka Korobowicz-Markiewicz**

Poland

**Magdalena Kucia**

USA

**Emmanuel Laffitte**

Switzerland

**Pekka Lahdenne**

Finland

**Carol Landis**

USA

**Thomas JA Lehman**

USA

**Valentina Leone**

United Kingdom

**Suzanne Li**

USA

**Kimberly Liang**

USA

**Carol Lindsley**

USA

**Worawit Louthrenoo**

Thailand

**Daniel Lovell**

USA

**Veronica Lundberg**

Sweden

**Claudia Macaubas**

USA

**Maria Cristina Maggio**

Italy

**Joanna Makowska**

Poland

**Bernhard Manger**

Germany

**Liza McCann**

United Kingdom

**Michael Miller**

USA

**Kirsten Minden**

Germany

**Nobuyuki Miyasaka**

Japan

**Yikun Mou**

China

**Kiran Nistala**

United Kingdom

**Sheila Oliveira**

Brazil

**Kathleen O'Neil**

USA

**Karen Onel**

USA

**Marina Ostroukhova**

Uruguay

**Ferda Ozkinay**

Turkey

**Anjali Patwardhan**

USA

**Sampath Prahalad**

USA

**Athimalaipet Ramanan**

United Kingdom

**Angelo Ravelli**

Italy

**Graham J Reid**

Canada

**Andreas Reiff**

USA

**Angela Robinson**

USA

**Alan Rosenberg**

Canada

**Margalit Rosenkranz**

USA

**Johannes Roth**

Germany

**Ricardo Russo**

Argentina

**Marite Rygg**

Norway

**Claudia Saad-Magalhães**

Brazil

**Rotraud Saurenmann**

Switzerland

**Ken Schikler**

USA

**Heinrike Schmeling**

Canada

**Kara Murphy Schmidt**

USA

**Rayfel Schneider**

Canada

**Kelsey Smith**

USA

**Clayton Sontheimer**

USA

**Betul Sozeri**

Turkey

**Steven Spalding**

USA

**Anne Helene Spannow**

Denmark

**Elizabeth Stringer**

Canada

**Hiroshi Tanaka**

Japan

**Tracy Ting**

USA

**Troy Torgerson**

USA

**Karine Toupin April**

Canada

**Marinka Twilt**

Canada

**Nikolay Tzaribachev**

Germany

**Yosef Uziel**

Israel

**Janjaaap van der Net**

The Netherlands

**Sebastiaan Vastert**

The Netherlands

**Richard Vehe**

USA

**Jelena Vojinovic**

Serbia

**Carol Wallace**

USA

**Toru Watanabe**

Japan

**Pamela Weiss**

USA

**Patricia Woo**

United Kingdom

**Nico Wulffraat**

The Netherlands

**Shuang Ye**

China

**Francesco Zulian**

Italy

